# To “Regulate the Practice”

**Published:** 1895-03

**Authors:** 


TO "REGULATE THE PRACTICE."—The Texas profession is still alive to the necessity of making an effort to secure better legislation in the interest of the public; and although every effort, so far, has failed, they seem to be not discouraged, but determined to renew their exertions. In our February number, we gave an outline of the bill introduced by Dr. Wooten. At this writing, it has not passed. There is at the same time now pending in the legislature a mongrel bill, introduced by whom we know not, which provides for *four examining boards*, including one for the "physico-medicals," whatever that may be.

The Houston District Medical Society are moving in the matter, and have sent out a request to all the local Texas Medical Societies to co-operate with them, and to memorialize the respctive county representatives in the legislature on the subject. They have set the example themselves, by issuing the following memorial. Ex-President E. T. Cook was sent up to Austin by the Houston Society, as delegate, instructed to push the memorial by personal interviews with the members:

MEMORIAL.

The Houston District Medical Association memorializes our representatives in the legislature, praying them to procure an amendment to the act to Regulate the Practice of Medicine in the State of Texas, to the effect that all that part of the law requiring or permitting any one to register a diploma, be eliminated, so that any one desiring to practice medicine in the State of Texas shall first procure and register with the clerk of the district court of the district in which such person may reside or sojourn, *a certificate of proficiency from some accredited* medical examining board in the State of Texas, under penalties prescribed by the Penal Code of the State of Texas.

Yours truly,

S. C. RED, M. D.,

President, Houston District Medical Association.

ROBT. T. MORRIS, M. D., Secretary.

